# Targeted Next Generation Sequencing to study insert stability in genetically modified plants

**DOI:** 10.1038/s41598-019-38701-9

**Published:** 2019-02-19

**Authors:** Anne-Laure Boutigny, Audrey Barranger, Claire De Boisséson, Yannick Blanchard, Mathieu Rolland

**Affiliations:** 1 Anses, Plant Health Laboratory, Bacteriology Virology GMO Unit, 7 rue Jean Dixméras, 49044 Angers cedex 01, France; 2 Anses, Ploufragan Laboratory, Viral Genetics and Biosafety Unit, BP 53, 22440 Ploufragan, France

## Abstract

The EU directive 2001/18/EC requires any genetically modified (GM) event to be stable. In the present work, a targeted Next-Generation Sequencing (NGS) approach using barcodes to specifically tag each individual DNA molecules during library preparation was implemented to detect mutations taking into account the background noise due to amplification and sequencing errors. The method was first showed to be efficient in detecting the mutations in synthetic samples prepared with custom-synthesized mutated or non-mutated P35S sequences mixed in different proportions. The genetic stability of a portion of the P35S promoter targeted for GM detection was then analyzed in GM flour samples. Several low frequency mutations were detected in the P35S sequences. Some mutated nucleotides were located within the primers and probes used in the P35S diagnostic test. If present not as somatic mutations but as the consensus sequence of some individuals, these mutations could influence the efficiency of the P35S real time PCR diagnostic test. This methodology could be implemented in genetic stability studies of GM inserts but also to detect single nucleotide mutant GM plants produced using “new breeding techniques”.

## Introduction

Since the first year of commercialization in 1996, the development of genetically modified (GM) crops has constantly grown in terms of cultivated land area, plant species and diversity of GM characteristics. In 2017, 24 countries grew 189.8 million hectares of GM crops worldwide (mostly soybean, maize, cotton, and canola)^1^. From 1992 to 2017, 4,133 approvals were issued by regulatory authorities for 29 GM crops and 498 events and this number is expected to rise^1^. Approvals were issued to GM crops for food use, feed use, and environmental release or cultivation.

According to the EU Directive 2001/18/EC on the deliberate release into the environment of genetically modified organisms and repealing Council Directive 90/220/EEC^2^, approval of a new GM crop requires the GM inserts to be genetically characterized and quantified. Applicants must provide information concerning genetic stability of the insert in their notifications. Development, cultivation and propagation of the GM crops should not induce any changes in the introduced DNA sequence^3^. For plant containing single transformation events, genetic stability encompassing the Mendelian inheritance of the insert and the molecular stability of the event over multiple (usually five) generations or vegetative cycles has to be demonstrated^2,4^. The Mendelian inheritance is currently checked by segregation analysis and the Chi-square test^4^. Molecular stability of the event is commonly verified by Southern Blot analyses; however this technique is not appropriate for detecting small DNA changes^5^ and sequencing approaches represent interesting alternatives^4^. The detection of small DNA rearrangements is important as one or more nucleotide polymorphisms in the insert could potentially modify transgene expression eventually leading to the loss of the GM characteristics^6–8^. Aguilera *et al*. (2008) verified the genetic stability of the MON810 trait in seeds of 26 commercial maize varieties by analyzing the intactness of MON810 integration using PCR-RFLP. The MON810 insert was absent in one maize variety suggesting the loss of the transgene locus. In addition to transgene expression alteration or loss, GM detection and quantification methods based on real-time PCR could also be affected by DNA changes. The presence of GMOs is currently assessed by reference laboratory *via* a screening approach targeting the most common transgenic elements found in GMOs, such as the Cauliflower mosaic virus (CaMV) 35S promoter (P35S) and the nopaline synthase terminator from *Agrobacterium tumefaciens* (tNOS)^9^. Once the presence of GM material has been established using screening methods, GMOs are further identified using GM event specific real time PCR methods. Nucleotide polymorphisms within primer and/or probe sequences could alter the method’s reliability, the position of single-nucleotide polymorphisms (SNPs) within oligonucleotides influencing their melting temperature (Tm), the efficiency of polymerase extension, and target specificity^10^.

Some published studies have investigated the post-transformational stability of DNA inserts and their flanking regions in Roundup Ready (RR) soybean^7^, MON810 maize^6,8,11,12^, stacked maize MON88017 x MON810^3^ and NK603 x MON810^5,12^ and GT73 oilseed rape^3^. In 2005, Ogasawara *et al*. studied frequency of mutations of the transgene in 72 Roundup Ready (RR) soybean seeds by sequencing 572 bp PCR fragments of the transgene and identified several mutated nucleotides in the 35S promoter region. Ben Ali *et al*. (2014; 2018) detected some variants in the *cry1Ab* coding region of seed samples of MON810 maize varieties. They also reported that samples from seeds containing a stacked MON810 event had more variants than MON810 single event varieties^12^. The more recent studies used a combination of real-time PCR, High-Resolution Melting (HRM) analyses, Sanger sequencing and Illumina sequencing which are appropriate techniques able to identify small DNA rearrangements^3,5,12^. Real-time PCR coupled with HRM is a rapid, simple, robust and sensitive method for detecting mutation and enabling the screening of many samples^13^. Nevertheless, this method does not provide information about nucleotide sequences and requires subsequent sequencing. In addition, HRM being based on the melting properties of the double stranded DNA molecules synthetized during PCR amplification, the obtained result is characteristic of the consensus sequence of the DNA molecules present in the sample and does not provide information on the variability of the DNA sequences within the sample. It is therefore only suitable for the analysis of separate individuals (seeds or plants). Rare mutations cannot be detected by analyzing individuals one by one. Their detection requires the simultaneous analysis of a large population of individuals ideally coming from different environments. Next Generation Sequencing (NGS) approaches can provide the sequence of numerous DNA molecules present in an extract. For these reasons, the application of NGS approaches on DNA extracts from flour, which are obtained from populations of seeds from various plants and environments, constitutes a method of choice to characterize the stability of a GM insert.

Currently, the targeted NGS approach is extensively used to identify mutations in various complex samples^14–18^. However, the detection of rare mutations (frequency below 1%) by NGS remains challenging because of the background noise due to errors introduced by the polymerase during library preparation and to the error rate of sequencing technologies^19–21^. In recent years, effective methods for detecting rare mutations have been developed and investigated for clinical and research applications including non-invasive prenatal diagnostics, early detection of cancer, monitoring of treatment response, detection of infectious disease and food safety^19,22,23^. Several groups have described barcode-based molecular approach to effectively distinguish amplification and sequencing errors from true mutations^19,20,22–27^. During library preparation, each individual DNA molecule present in the sample is tagged by incorporating a specific molecular barcode^14^. After amplification of the tagged DNA, multiple copies of each molecule are sequenced and reads with the same molecular barcode are grouped into families to create a high confidence consensus^14,23^. Mutations found in only a fraction of the reads with the same barcode are amplification or sequencing errors and are eliminated when the consensus is created^23^. Mutations found in all the reads with the same barcode are true mutations. Stalhberg *et al*. (2016, 2017) developed a barcode-based approach called “Simple, Multiplexed, PCR-based barcoding of DNA for Sensitive mutation detection using Sequencing” (SiMSen-Seq). Their method uses reduced primer concentration, elongated PCR extension times and hairpin-protected barcode primers to enable simple and short library construction protocol^20^. SiMSen-Seq can detect variants at or below 0.1% frequency with low DNA input. A detailed protocol for assay development and a bioinformatics package to process the barcoded data are described in Stalhberg *et al*. (2017). For these reasons, SIMSen-Seq was implemented in our laboratory to study GM insert stability in complex samples. The methodology was first assessed on synthetic samples prepared with custom-synthesized mutated or non-mutated sequences mixed in different proportions. The variability of a portion of the P35S promoter targeted for GM detection was then analyzed in GM flour samples.

## Results

### Assay quality

In our assay, 1,329,877 (natural sample 1) to 2,095,571 (synthetic sample 10% C4) raw reads were obtained with a mean of 1,883,581 raw reads in synthetic samples and 1,749,361 in natural samples (Tables 1 and 2). Among these raw reads, 1,262,132 (natural sample 1) to 2,080,079 (synthetic sample 10% C4) amplicon reads were identified with a mean of 1,869,150 amplicon reads in synthetic samples and 1,703,026 in natural samples. Amplicon yields (amplicon reads/raw reads) ranged from 0.95 to 0.99, indicating that a very high number of the total data were analyzable. The number of consensus sequences identified per sample is presented according to consensus depth (3, 10, 20 or 30), which corresponds to the number of raw reads with the same barcode used to compute a consensus sequence. The mean number of consensus sequences identified was 199,506 for synthetic samples and 67,541 for natural samples using a consensus(3), 67,857 (synthetic samples) and 39,666 (natural samples) using a consensus(10), 7,187 (synthetic samples) and 24,269 (natural samples) using a consensus(20) and 737 (synthetic samples) and 17,407 (natural samples) using a consensus(30). The distribution of barcode family depths was larger for the natural samples than for the synthetic samples (Supplementary Figure S1). Down-sampling plots (Supplementary Figure S2) showed a saturation of detected barcodes in the synthetic samples (visible horizontal plateau) while few additional barcodes could have been detected in the natural samples (light upward slope) with additional sequencing depth. Overall, summary statistics showed our assay to be of very good quality.

**Table 1:** Debarcer summary statistics for synthetic samples.

Debarcer summary statistics	Synthetic samples						
	10% C1	1% C1	0.1% C1	10% C4	1% C4	0.1% C4	mean
Raw reads	1,967,009	1,768,030	1,770,867	2,095,571	1,816,428	1,770,867	1,883,581
Amplicon reads identified	1,952,977	1,753,726	1,755,685	2,080,079	1,803,282	1,755,685	1,869,150
Amplicon yield	0.99	0.99	0.99	0.99	0.99	0.99	0.99
Consensus (3) reads	211,478	177,379	200,867	207,975	199,829	200,867	199,506
Consensus (10) reads	70,454	68,185	57,655	81,023	61,966	57,655	67,857
Consensus (20) reads	6,874	8,364	4,733	10,363	5,601	4,733	7,187
Consensus (30) reads	628	989	352	1,220	495	352	737

**Table 2:** Debarcer summary statistics for natural samples.

Debarcer summary statistics	Natural samples						
	1	2	3	4	5	6	mean
Raw reads	1,329,877	1,853,807	1,579,627	1,877,569	1,672,575	1,763,225	1,749,361
Amplicon reads identified	1,262,132	1,821,157	1,568,245	1,844,744	1,609,341	1,671,643	1,703,026
Amplicon yield	0.95	0.98	0.99	0.98	0.96	0.95	0.97
Consensus (3) reads	44,449	42,608	160,142	67,629	43,832	23,492	67,541
Consensus (10) reads	32,811	34,407	56,641	52,770	35,314	19,199	39,666
Consensus (20) reads	24,270	28,699	7,807	37,972	29,563	17,305	24,269
Consensus(30) reads	16,062	24,511	717	23,391	22,416	16,001	17,407

### Analysis of mutation in synthetic samples

**Figure 1:**
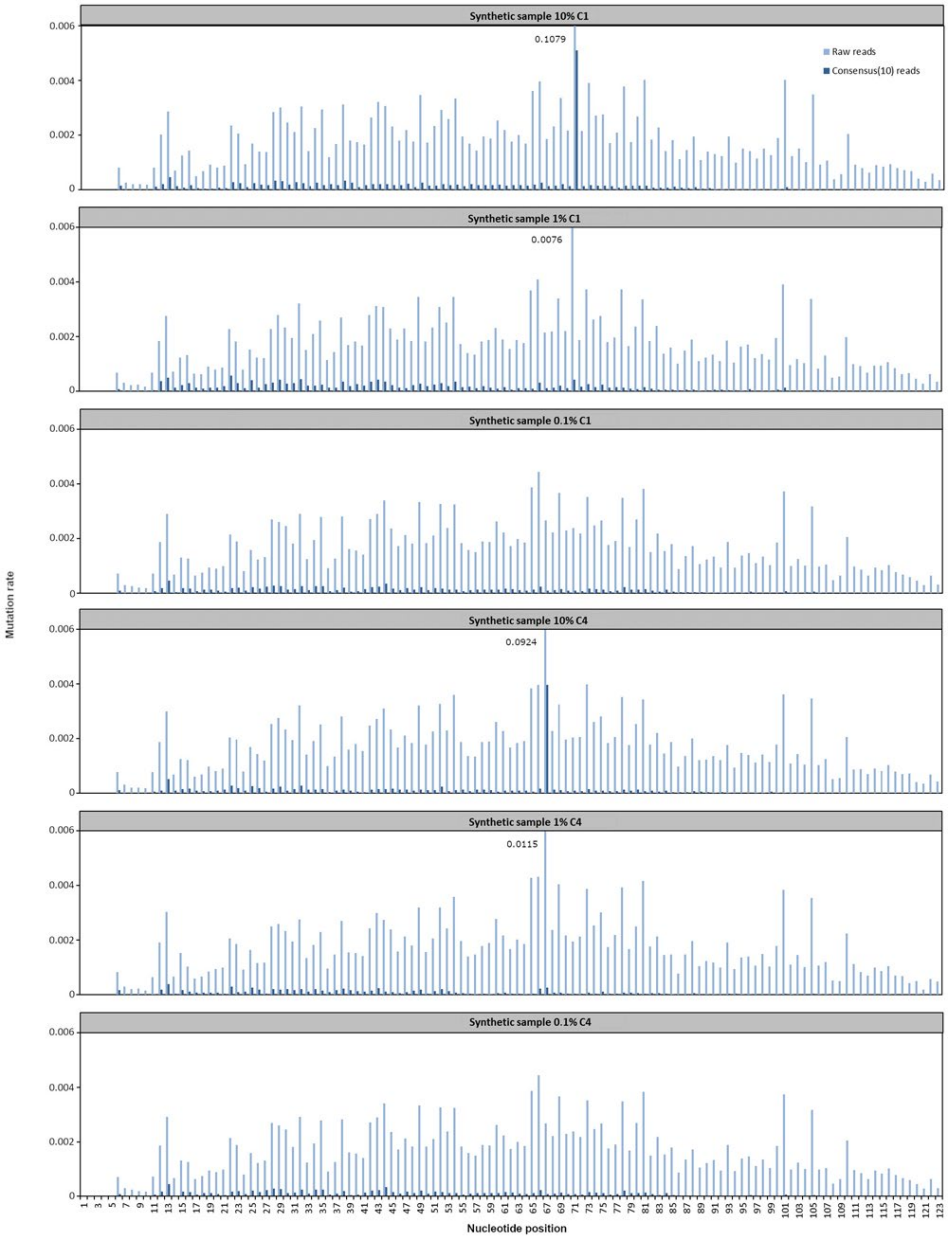
Raw and corrected mutation rates at each position of the P35S sequence studied in the synthetic samples. 10% C1, 1% C1, 0.1% C1: synthetic samples containing 10%, 1% or 0.1% of the P35S-C1 oligonucleotide mixed with respectively 90%, 99% or 99.9% of the P35S-WT oligonucleotide. 10% C4, 1% C4, 0.1% C4: synthetic samples containing 10%, 1% or 0.1% of the P35S-C4 oligonucleotide mixed with respectively 90%, 99% or 99.9% of the P35S-WT oligonucleotide.

Using Debarcer, raw mutation rates and corrected mutation rates (consensus(3), consensus(10), consensus(20), consensus(30)) were obtained at each position of the P35S sequence studied in the synthetic samples. The raw and corrected consensus(10) mutation rates are presented in Fig. 1. All along the sequence (excluding positions 67 and 71), mutations were detected with a mean mutation rate of 0.165% using raw reads, 0.026% using consensus(3) reads (data not shown) and 0.010% using consensus(10) reads and a maximum error rate of 0.44% using raw reads, 0.1% using consensus(3) reads (data not shown) and 0.05% using consensus(10) reads. Mutations detected at other positions than 67 and 71 correspond to background noise which could be associated with the long oligonucleotide synthesis error rate. The mean mutation rate of 0.010% using consensus(10) reads corresponds to the announced synthesis error rate of 0.050–0.066% (according to the provider, Eurofins).

Mutation rates at positions 67 and 71 are presented in Table 3 using raw reads and corrected consensus(3) or consensus(10) reads. Taking into account the maximum background noise (0.44% using raw reads, 0.1% using consensus(3) reads and 0.05% using consensus(10) reads), the mutated nucleotide at position 67 was detected in sample 10% C4 at a rate of 9.04% using raw reads, 1.48% using consensus(3) reads and 0.36% using consensus(10) reads and in sample 1% C4 at a rate of 0.97% using raw reads and 0.14% using consensus(3) reads. Similarly, the mutated nucleotide at position 71 was detected in sample 10% C1 at a rate of 10.65% using raw reads, 1.99% using consensus(3) reads and 0.48% using consensus(10) reads and in sample 1% C1 at a rate of 0.59% using raw reads. The raw mutation rate was close to the real percentage of mutated sequence in the samples, while the corrected mutation rate was under-estimated. At positions 67 and 71, other mutations than the expected mutations were observed at low level (Table 3), corresponding to the synthesis error rates previously observed all along the sequence.

**Table 3:** Mutation rates (%) at positions 67 and 71 of the P35S sequence using raw reads and corrected consensus(3) or consensus(10) reads.

Position 67	Mutation A → T			Other mutations
	10% C4	1% C4	0.1% C4	mean
Raw reads	9.0386	0.9705	0.0599	0.1984
Consensus (3) reads	1.4797	0.1426	0.0065	0.0413
Consensus (10) reads	0.3555	0.0226	0.0000	0.0175
**Position 71**	**Mutation A → G**			**Other mutations**
	**10% C1**	**1% C1**	**0.1% C1**	**mean**
Raw reads	10.6484	0.5923	0.0649	0.1626
Consensus (3) reads	1.9858	0.1066	0.0080	0.0318
Consensus (10) reads	0.4841	0.0308	0.0000	0.0158

### Analysis of mutation in GM flour samples

**Figure 2:**
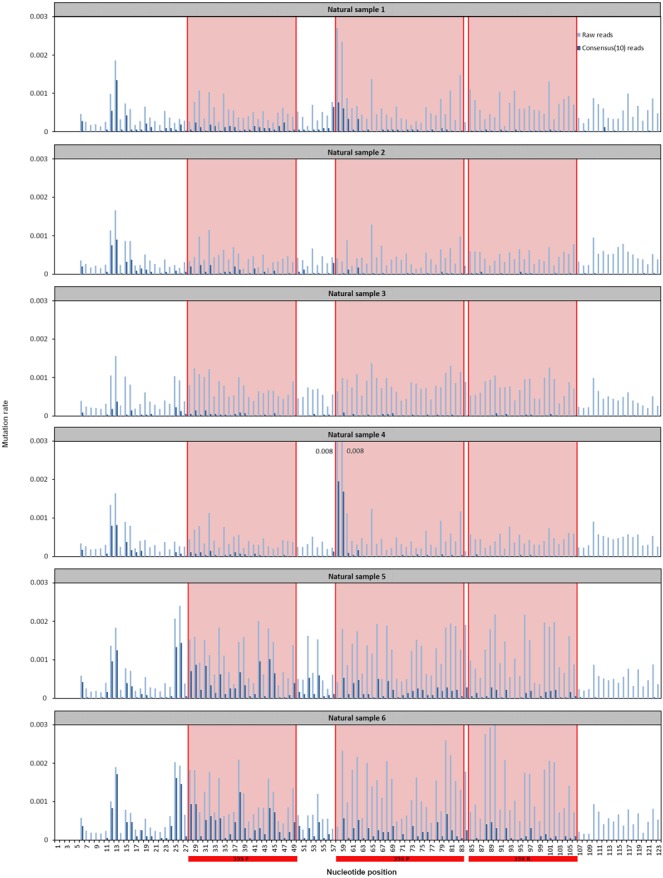
Raw and corrected mutation rates at each position of the P35S sequence studied in the natural samples. NS 1, NS 2, NS 3, NS 4, NS 5 and NS 6: Natural GM flour samples 1 to 6. 35SF, 35SR and 35SP: Primers F and R and probe P used to detect P35S using real-time PCR.

Using Debarcer, raw mutation rates and corrected error rates (consensus(3), consensus(10), consensus(20), consensus(30)) were obtained at each position of the P35S sequence studied in the natural samples. Results were analyzed using a corrected consensus depth of 10 as recommended previously^19^ (Fig. 2). At all positions, the corrected mutation rates were lower than the raw mutation rates. The correction applied on raw mutation rates varied depending on the individual nucleotide. In the corrected consensus(10) data, several mutated positions were identified all along the P35S sequence studied in the six samples. Mutation rates varied between the positions and samples, with a maximum of 0.2% at position 58 and 0.17% at position 59 in sample 4. In sample 3, mutation rates were below 0.05%. In other maize samples, mutation rates above 0.05% were observed at positions 12–13 for samples 1, 2 and 4, positions 57-58-59 for sample 1 and positions 58–59 for sample 4. Overall, mutation rates were slightly higher in soybean flours, with 16 positions with mutation rates above 0.05% in samples 5 (at positions 12, 13, 25, 26, 28, 29, 31, 34, 38, 42, 44, 45, 52, 54, 59, 66) and 6 (at positions 12, 13, 25, 26, 28, 29, 31, 32, 33, 34, 38, 44, 45, 59, 62, 80).

## Discussion

In this study, a new approach using targeted NGS detected several low frequency mutations in a portion of P35S sequences in complex GM samples. The SiMSen-seq methodology had previously been used for ultrasensitive mutation detection in liquid biopsies^20^ and tumors^28^. This was the first time this methodology was successfully implemented to detect mutations in plant materials.

In order to test the technique in our laboratory, synthetic samples were prepared and analyzed, containing custom-synthesized mutated or non-mutated P35S sequences mixed in different proportions. The method was efficient in detecting the mutations introduced in the P35S sequence regardless of the position in the sequence or the class of mutation (class I or IV). Custom-synthesized sequences were good materials to test the efficiency of the primers and the method in our laboratory but they could not be used to evaluate the sensitivity of the assay below 1% because of the synthesis error rate creating a background noise. Nevertheless, the method could detect mutations below 0.05% in GM flour samples, indicating a very good sensitivity of the assay.

In our study, hundreds of thousands of P35S sequences extracted from complex GM samples such as maize and soybean seed flours were analyzed individually. This constitutes a major improvement compared to techniques used recently (HRM techniques coupled with sequencing) which analyzed individual seeds one by one^3,5,12^. By providing the sequence of many individual DNA molecules present in the sample, this methodology allows the analysis of a population of individuals, but it also provides an insight into the mutations which occurred within individuals. The drawback associated with the power of the technique is that some of the mutations observed in the results may have been present in somatic cells without being present in the consensus sequence of any individuals.

**Figure 3:**
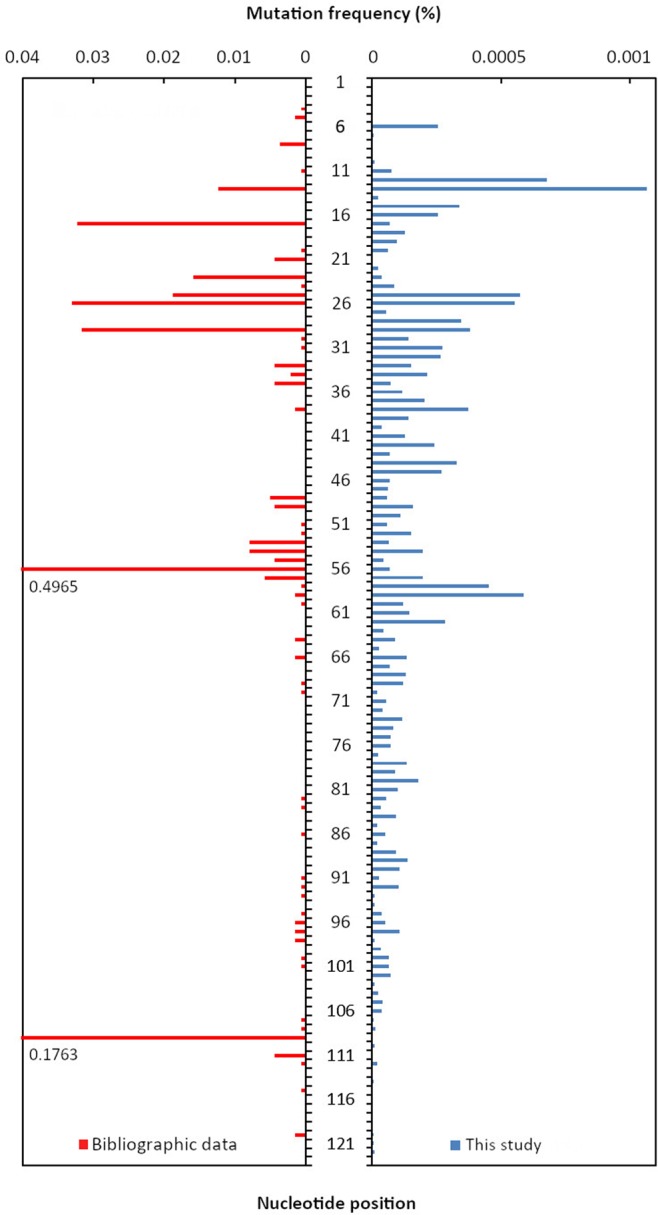
Mutation frequencies at each position of the P35S sequence in bibliographic data and in this study. In bibliographic data, 2,746 sequences were retrieved from GenBank after blasting our target P35S sequence (nr/nt, megablast, at least 70% coverage and 90% homology). The 2,746 sequences were mapped to our target sequence using Geneious and mutation frequency was calculated at each position of the P35S sequence. In this study, the presented data correspond to the mean mutation frequencies of the 6 natural samples studied.

Because of its high expression level, the 35S promoter is the most popular and common promoter of transgenes in GM plants^7^. In transgenic plants, the P35S promoters come from various strains of Cauliflower mosaic virus and have been introduced *via* different vector constructions^29^. For these reasons, there may be polymorphisms in the sequences of the P35S promoter. In addition, mutations may have occurred during the breeding process^29^. This study focused on the conserved region of the P35S promoter targeted by diagnostic methods using real-time PCR. Several mutated nucleotides were identified, with frequency varying between 0.2% to less than 0.05%. By analyzing bibliographic data, 2,746 P35S sequences were retrieved from GenBank database and mapped to our target sequence. At each position of the sequence of interest, mutation frequencies from bibliographic data and from this study are presented in Fig. 3. The frequency of mutations found in the published data does not truly correspond to the mutation rate of the sequence. Indeed this frequency may over represent mutations contained in cloned P35S promoters, which have been extensively sequenced. On the opposite, the data presented by this study truly correspond to the mutation rate in the analyzed samples. Several SNPs were newly identified during this study while some can be retrieved in previously published data. Some of the identified mutated nucleotides are located within the primers and probes used in the P35S diagnostic test, the forward primer being located between nucleotides 28 and 49, the probe between nucleotides 58 and 83 and the reverse primer between nucleotides 85 and 106 (Fig. 2). The mutation frequency (below 0.2%) will likely not influence the efficiency of the P35S real time PCR diagnostic test. Nevertheless, such samples (mixed flour with several GM events) can indicate mutated positions that could be more specifically looked at in flours made with single GM events. Ghedira *et al*.^30^ assessed primer/template mismatch effects on real-time PCR amplification for GMO quantification. They showed that a mismatch between the primer and the DNA template can cause partial to complete failure of the amplification of the initial DNA template depending on the type and location of the nucleotide mismatch affecting the estimated total DNA quantity to a varying degree^30^. Morisset *et al*. (2009) identified a SNP in the primer binding site of the P35S sequence in maize TC1507, leading to inefficient amplification^31^. In another study, Lefever *et al*. (2013) showed that four mismatches in a single primer can block amplification almost completely, whereas three mismatches in one of the primers must be combined with at least two mismatches in the other primer to achieve the same extent of inhibition^10^.

In addition to an alteration of the real-time PCR diagnostic test, mutated nucleotides in the promoter sequence could potentially influence its activity and consequently the expression rate of the transgene^5^. Several DNA sequences or motifs are required for its activity^32–36^. Mutations occurring in essential DNA sequences, such as the boxes for transcription factors CAAT and TATA, could stop or reduce promoter activity, which could potentially lead to the loss of the GM characteristic. Spontaneous mutations can occur in all living organisms during DNA replication, homologous or intrachromosomal recombination, mitosis and meiosis^11,12^. The mutation rate of maize genes has been estimated at 3 × 10^−8^ substitutions per site and per generation^37^. It has been suggested that once integrated into the genome, the rate of mutation of transgenes should be the same as that of internal host genes^6,7^. Ogasawara *et al*. (2005) compared the mutation rate of the transgene for RR soybean and an internal host gene, the Cong gene, and obtained almost the same mutation rate. Point mutations such as small deletions or substitutions can also occur as a response of the plants to environmental conditions^38^.

In this study, the SiMSen-seq methodology was successfully implemented for sensitive mutation detection in P35S sequences in complex GM samples. The method presents a significant improvement to detect variants in GM inserts due to its sensitivity below 0.05% and because it addresses the main limitation of the use of NGS for mutant detection (i.e. amplification and sequencing errors). In addition, the present study looks for the presence of mutants in a population of DNA extracted from complex samples which seems preferable to the analyses of individual seeds one by one. By analyzing flour complex samples, it proves that the methodology works well and several mutated positions were even reported in the P35S sequence. Because the flour samples contained several GM events, it was not possible to associate some SNPs to a particular GM event. It would be interesting to separately analyze flours made by single GM events to investigate if some events show a more abundant mutation rate compared to others.

In conclusion, the present study focused on a portion of the P35S sequence targeted by screening methods used to undertake a first assessment of the GMO status of samples. Its genetic stability is important for the reliable control of GMOs, food labelling and the traceability of GM plants. This approach could be used to explore the mutation rate in other screening GM targets like the nopaline synthase terminator from *Agrobacterium tumefaciens* (tNOS), the phosphinothricin acetyltransferase gene from *Streptomyces hygroscopicus* (bar) or the 5-enolpyruvylshikimate-3-phosphate synthase gene from *Agrobacterium tumefaciens* sp. strain CP4 (ctp2-cp4epsps). In addition, this method could analyze several GM target at the same time due to its multiplexing capacity. Finally, associated with long-read sequencing instruments, this strategy could cover GM inserts of several kilobases and could be implemented in genetic stability studies or could be used to detect single nucleotide mutant GM plants produced using “new breeding techniques”.

## Materials and Methods

### Materials

P35S oligonucleotides (5′-TCGTGGAAAAAGAAGACGTTCCAACCACGTCTTCAAAGCAAGTGGATTGATGTGATATCTCCACTGACGTAAGGGATGACGCACAATCCCACTATCCTTCGCAAGACCCTTCCTCTATATAAGGAAGTTCAT-3′; 132 bp), modified or not, were custom synthesized by Eurofins (Luxembourg). The P35S-WT oligonucleotide had no SNP. The P35S-C1 oligonucleotide had a class I SNP (A → G) at position 71 and the P35S-C4 oligonucleotide had a class IV SNP (A → T) at position 67. Oligonucleotides were resuspended in TE buffer (Tris, 10 mM; EDTA, 1 mM; pH 8) at 100 pmol/µL. They were diluted in 0.1X TE buffer in order to prepare synthetic samples by mixing P35S-WT, P35S-C1 and P35S-C4 oligonucleotide solutions in different proportions at a final concentration of 160,000 cp/µL. Six synthetic samples were prepared: 10% C1 (10% of the P35S-C1 oligonucleotide mixed with 90% of the P35S-WT oligonucleotide), 1% C1 (1% of the P35S-C1 oligonucleotide mixed with 99% of the P35S-WT oligonucleotide), 0.1% C1 (0.1% of the P35S-C1 oligonucleotide mixed with 99.9% of the P35S-WT oligonucleotide), 10% C4 (10% of the P35S-C4 oligonucleotide mixed with 90% of the P35S-WT oligonucleotide), 1% C4 (1% of the P35S-C4 oligonucleotide mixed with 99% of the P35S-WT oligonucleotide) and 0.1% C4 (0.1% of the P35S-C4 oligonucleotide mixed with 99.9% of the P35S-WT oligonucleotide). Synthetic samples were analyzed to test the efficiency of the technique for detecting known mutations.

Six natural flour samples (NS1, NS2, NS3, NS4, NS5 and NS6) containing several GM events were obtained from the Joint Laboratories Service Unit in Strasbourg (France). These samples tested positive for P35S using real-time PCR (data not shown). Samples 1 to 4 mainly contained maize while samples 5 and 6 mainly contained soybean. These natural flour samples were analyzed to test the efficiency and sensitivity of the technique for detecting unknown mutations.

In the manuscript, natural samples refer to GM flour samples in opposition to synthetic samples that refer to samples prepared by mixing custom synthesized sequences of P35S.

### DNA extraction

Genomic DNA was extracted from flour using a cetyltrimethylammonium bromide (CTAB) method. 250 mg of flour were suspended in 1.2 mL of CTAB extraction buffer (CTAB, 20 g/L; NaCl, 1.4 M; Tris-HCl, 0.1 M; Na2EDTA, 20 mM, pH 8.0) preheated to 65 °C. A volume of 10 µL of α-amylase solution (10 mg/mL) and a volume of 10 µL of RNase (10 mg/mL) were added and the mixture was incubated at 65 °C for 30 min, with vortex mixing every 10 min. A volume of 20 µL of proteinase K (20 mg/mL) was added and the mixture was incubated at 65 °C for another 30 min, with vortex mixing every 10 min. After centrifugation at 12,000 g for 10 min, the supernatant was transferred to a new 2 mL microtube with 240 µL of ammonium acetate (10 M) and 600 µL of isopropanol, mixed by inverting the tube and centrifuged at 12,000 g for 15 minutes. The supernatant was discarded and resulting pellets were dissolved in 200 µL of TE buffer. DNA were purified using the QIAquick PCR Purification Kit (Qiagen, Hilden, Germany) according to the manufacturer’s instructions except that they were eluted in 100 µL of Buffer EB. DNA concentrations were measured with the Qubit 2.0 fluorometer (Life Technologies, Carlsbad, United States) and DNA were stored at −20 °C.

### Target primer design

The P35S sequences of several GM plants were retrieved from GenBank or the GMDD database. The sequences were aligned using CLUSTALW available in Geneious 11.0.5. Two sets of primers were designed using PRIMER 3 available in Geneious to amplify the sequence of P35S traditionally used in real time PCR analyses^39^. The primers were tested as previously recommended^19^. Primers P35S-F2 (5′-TCGTGGAAAAAGAAGACGTTCC-3′) and P35S-R1 (5′-ATGAACTTCCTTATATAGAGGAAGGG-3′) were selected for the following experiments. The PCR product size was 132 bp. Barcode PCR primers (‘barcode forward primer’ and ‘barcode reverse primer’, see^19^) were ordered (PAGE-purified, Eurogentec, Liège, Belgium) and stock solutions were prepared as described previously^19^.

### Mutation analysis

DNA barcoding was performed by PCR in 10 µL using 1X AccuPrime PCR Buffer II, 0.2 U AccuPrime Taq DNA Polymerase High Fidelity (Invitrogen, Thermo Fisher Scientific, Waltham, United States), 80 nM of each barcode primer, 0.05 mg/mL of BSA and 800,000 copies DNA (synthetic samples), 25,000 copies DNA for flour sample 1, 194,040 copies DNA for flour sample 2, 83,930 copies DNA for flour sample 3, 117,040 copies DNA for flour sample 4, 25,000 copies DNA for flour sample 5 and 25,000 copies DNA for flour sample 6. The temperature profile was 98 °C for 3 min followed by three cycles of amplification (98 °C for 10 sec, 62 °C for 6 min and 72 °C for 30 sec). A volume of 20 µL of protease (30 ng/µL in TE buffer) was added and the mixture was incubated at 65 °C for 15 min and 95 °C for 15 min. The second PCR was performed in 40 µL using 1X Q5 Hot Start High Fidelity Master Mix (New England BioLabs, Ipswich, United States), 400 nM of each Illumina adaptor primer (NEBNext Multiplex Oligo for Illumina, index primers, New England Biolabs) and 10 µL of PCR products from the first PCR. Sequences of Illumina adaptor primer are presented in Stalhberg *et al*. (2016; 2017). The temperature profile was 95 °C for 3 min followed by 35 cycles of amplification (98 °C for 10 s, 80 °C for 1 s and 72 °C for 1 min). 40 µL (synthetic sample) or 120 µL (flour sample) of PCR products were purified using the Agencourt AMPure XP beads (Beckman Coulter, Brea, United States) according to the manufacturer’s instructions with a volume ratio between beads and PCR products of 1. The purified products were eluted in 20 µL (synthetic sample) or 45 µL (flour sample) of low TE buffer (10 mM Tris, 0.1 mM EDTA, pH 8.0). Validation of the librairies was obtained by capillary electrophoresis using the Fragment Analyzer and the High Sensitivity NGS fragment analysis kit (Advanced Analyticals, Evry, France). The librairies were stored at −20 °C prior to sequencing. Sequencing was performed on a MiSeq instrument (Illumina, San Diego, United States) at GenoBIRD (Nantes, France) using 150 pb paired-end reads. Raw FastQ files were processed using Debarcer, a package for De-Barcoding and Error Correction of sequencing data containing molecular barcodes, previously developed^19,20^. Raw data were deposited in the Sequence Read Archive under BioProject ID PRJNA475037.

## Supplementary information

Supplementary Figures
