# Supplementary material for: Targeted Next Generation Sequencing to study insert stability in genetically modified plants
Source: Sci Rep. 2019 Feb 19;9:2308. doi: 10.1038/s41598-019-38701-9 (PMC6381221; doi:10.1038/s41598-019-38701-9)
Supplement: Supplementary file 1 — Supplementary Figures [file 41598_2019_38701_MOESM1_ESM.pdf]

SREP-18-25447A

Targeted Next Generation Sequencing to study insert stability in genetically modified plants

Anne-Laure Boutigny[1,*], Audrey Barranger[1], Claire De Boisséson[2], Yannick Blanchard[2] and Mathieu Rolland[1]

Supplementary figures

Supplementary Figure 1

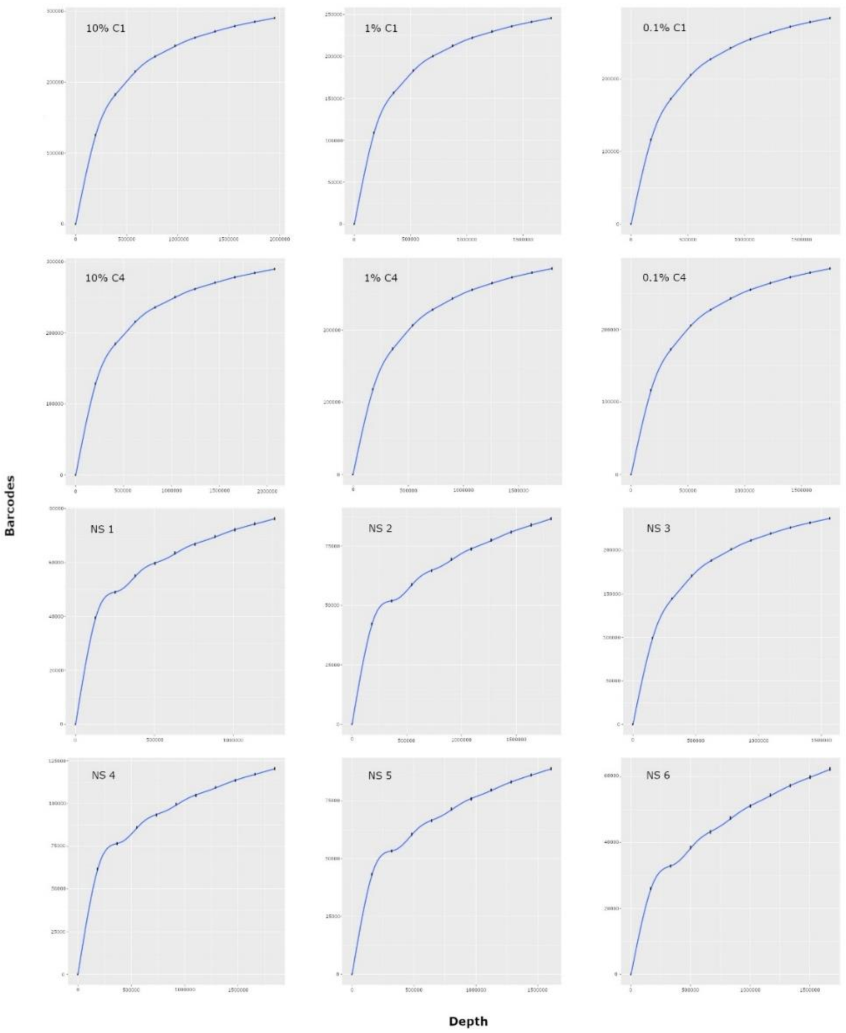

Supplementary Figure 1: Down-sampling plots produced by Debarcer.

10% C1, 1% C1, 0.1% C1: synthetic samples containing 10%, 1% or 0.1% of the P35S-C1 oligonucleotide mixed with respectively 90%, 99% or 99.9% of the P35S-WT oligonucleotide.

10% C4, 1% C4, 0.1% C4: synthetic samples containing 10%, 1% or 0.1% of the P35S-C4 oligonucleotide mixed with respectively 90%, 99% or 99.9% of the P35S-WT oligonucleotide.

NS 1, NS2, NS3, NS4, NS5 and NS6: Natural GM flour samples 1 to 6.

Supplementary Figure 2

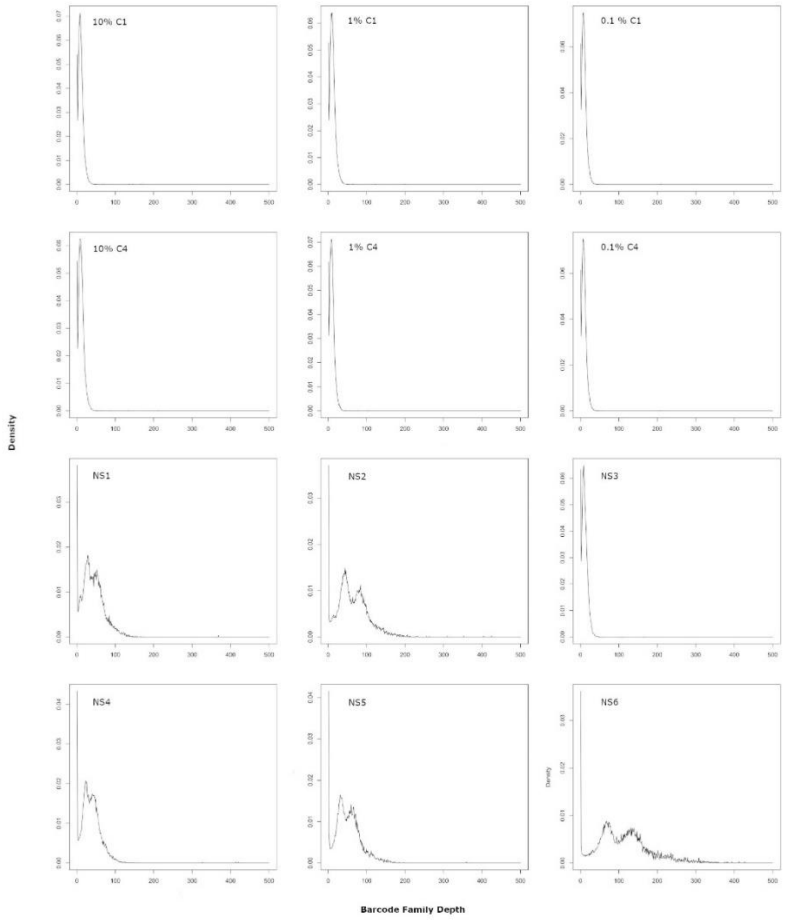

Supplementary Figure 2: Distribution of barcode family depths in synthetic and natural samples.

10% C1, 1% C1, 0.1% C1: synthetic samples containing 10%, 1% or 0.1% of the P35S-C1 oligonucleotide mixed with respectively 90%, 99% or 99.9% of the P35S-WT oligonucleotide.

10% C4, 1% C4, 0.1% C4: synthetic samples containing 10%, 1% or 0.1% of the P35S-C4 oligonucleotide mixed with respectively 90%, 99% or 99.9% of the P35S-WT oligonucleotide.

NS 1, NS2, NS3, NS4, NS5 and NS6: Natural GM flour samples 1 to 6.
